# Coordinated Activation of Candidate Proto-Oncogenes and Cancer Testes Antigens via Promoter Demethylation in Head and Neck Cancer and Lung Cancer

**DOI:** 10.1371/journal.pone.0004961

**Published:** 2009-03-23

**Authors:** Ian M. Smith, Chad A. Glazer, Suhail K. Mithani, Michael F. Ochs, Wenyue Sun, Sheetal Bhan, Alexander Vostrov, Ziedulla Abdullaev, Victor Lobanenkov, Andrew Gray, Chunyan Liu, Steven S. Chang, Kimberly L. Ostrow, William H. Westra, Shahnaz Begum, Mousumi Dhara, Joseph Califano

**Affiliations:** 1 Department of Otolaryngology—Head and Neck Surgery, Johns Hopkins Medical Institutions, Baltimore, Maryland, United States of America; 2 Department of Surgery, Division of Plastic and Reconstructive Surgery, Johns Hopkins Medical Institutions, Baltimore, Maryland, United States of America; 3 Division of Oncology Biostatistics, Department of Oncology, Johns Hopkins Medical Institutions, Baltimore, Maryland, United States of America; 4 Department of Pathology, Johns Hopkins Medical Institutions, Baltimore, Maryland, United States of America; 5 Institute of Allergy and Infectious Diseases, National Institute of Health, Rockville, Maryland, United States of America; 6 Milton J. Dance Head and Neck Center, Greater Baltimore Medical Center, Baltimore, Maryland, United States of America; City of Hope Medical Center, United States of America

## Abstract

**Background:**

Epigenetic alterations have been implicated in the pathogenesis of solid tumors, however, proto-oncogenes activated by promoter demethylation have been sporadically reported. We used an integrative method to analyze expression in primary head and neck squamous cell carcinoma (HNSCC) and pharmacologically demethylated cell lines to identify aberrantly demethylated and expressed candidate proto-oncogenes and cancer testes antigens in HNSCC.

**Methodology/Principal Findings:**

We noted coordinated promoter demethylation and simultaneous transcriptional upregulation of proto-oncogene candidates with promoter homology, and phylogenetic footprinting of these promoters demonstrated potential recognition sites for the transcription factor BORIS. Aberrant BORIS expression correlated with upregulation of candidate proto-oncogenes in multiple human malignancies including primary non-small cell lung cancers and HNSCC, induced coordinated proto-oncogene specific promoter demethylation and expression in non-tumorigenic cells, and transformed NIH3T3 cells.

**Conclusions/Significance:**

Coordinated, epigenetic unmasking of multiple genes with growth promoting activity occurs in aerodigestive cancers, and BORIS is implicated in the coordinated promoter demethylation and reactivation of epigenetically silenced genes in human cancers.

## Introduction

Epigenetic alterations in promoter methylation and histone acetylation have been associated with cancer-specific expression differences in human malignancies.

Methylation has been primarily considered as a mechanism of tumor suppressor gene (TSG) inactivation, and comprehensive whole-genome profiling approaches to promoter hypermethylation have identified multiple novel putative TSGs silenced by promoter hypermethylation.

Indirect evidence supports a role for *hypo*methylation in tumor development. Global genomic hypomethylation has been reported in almost all solid tumors [1]–[3]. Mice with functional disruption of DNA methyltransferase 1 (*DNMT1*) function demonstrate significant genomic hypomethylation in all tissues and develop aggressive T-cell lymphomas with chromosomal instability [4]. In solid human tumors, meta-analysis shows an overall correlation between global hypomethylation and advanced tumor stage[3].

To date, only sporadic examples of promoter hypomethylation associated with unmasked expression of putative oncogenes have been reported, including: *R-Ras* in gastric cancer [5], *c-Neu* in transgenic mouse models [6], the *Hox11* proto-oncogene in leukemia [7], *BCL-2* gene hypomethylation and high-level expression in B-cell chronic lymphocytic lymphomas [8], demethylation in MMTV/N-rasN transgenic mice [9], and rare activation of two *RAS* family members in colon cancer and small cell lung cancer [10]. These observations demonstrate that proto-oncogenes with tissue-specific or developmentally restricted expression—i.e., during early growth, differentiation, or gametogenesis—may be inappropriately re-expressed in cancers via epigenetic alteration, including demethylation.

HNSCC is useful as a solid tumor model system, due to the established role of epigenetic changes in its pathogenesis [11], as well as the availability of normal, minimally transformed cell lines for use in gene discovery strategies [12]. Using pharmacologic demethylation in normal, minimally-transformed oral keratinocyte cell lines combined with Cancer Outlier Profile Analysis (COPA) in primary tissues as a discovery approach, we were able to define a set of candidate proto-oncogenes that undergo aberrant demethylation and increased expression in primary human tumors.

Functional data and prior published observations suggest that expression of these genes is associated with tumor promotion. Additional analyses demonstrated promoter homology and coordinated upregulation in individual tumors for subsets of these target genes (proto-oncogenes). We were able to broaden these observations to a variety of solid tumor types and implicate a key transcription factor, BORIS, in coordinated epigenetic activation of proto-oncogenes. These data indicate that aberrant demethylation of multiple, physiologically repressed proto-oncogenes occurs in a coordinated fashion in individual tumors from multiple solid tumor types.

## Results

### Integrative Discovery of Epigenetically Unmasked Genes in HNSCC

We hypothesized that normal cell lines contain methylated genes that are typically repressed in normal tissues, but that these genes can be re-expressed by pharmacologic manipulation. A subset of these genes would include candidate proto-oncogenes activated by demethylation in human cancers that could be further selected on the basis of primary tumor expression array analysis using integrative methods. We chose to adapt prior methods of epigenetic screening using 5-aza/TSA treatment that have been found to be successful in defining candidate tumor suppressor genes. Two TERT-transformed normal oral keratinocyte cell lines were treated with 5 µM 5-aza deoxycytidine for four days and Trichostatin A for one day prior to harvesting total RNA for expression array analysis using dChip [12], [13].

**Figure 1:**
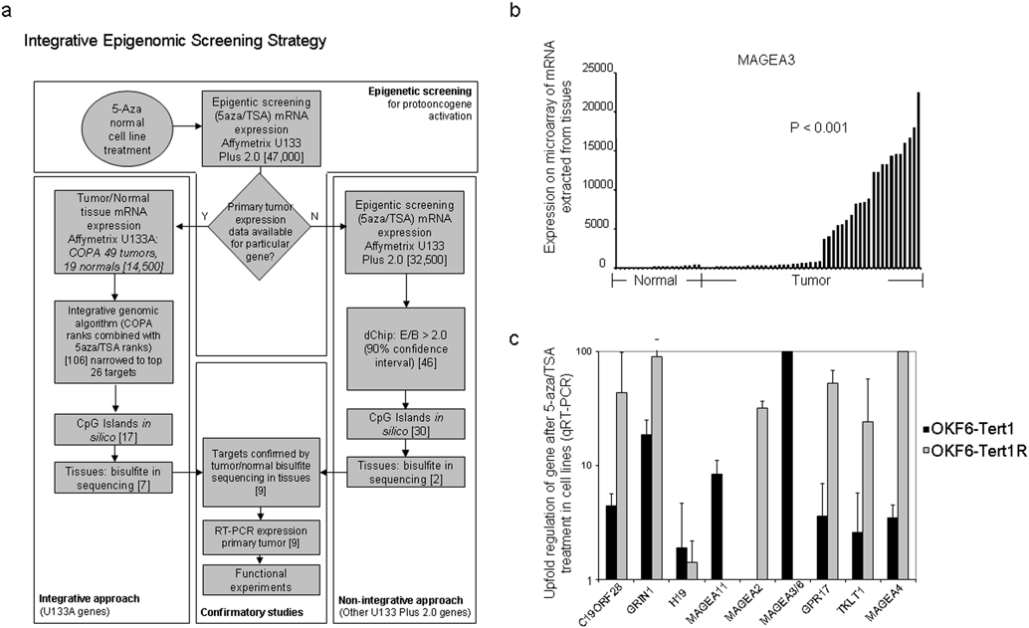
Integrative epigenetic screening strategy and strategy for validation of targets. (a) Initially, minimally-transformed cell lines were treated with 5-aza-deoxycytidine and TSA to unmask epigenetically silenced genes. In order to correlate epigenetic unmasking with meaningful upregulated cancer-specific genes, we performed a comparative epigenetic approach with Cancer Outlier Profiling Analysis (COPA) using 49 tumors and 19 normal tissues that had been characterized on the Affymetrix U133A mRNA expression microarray platform. Genes (by probeset) were ranked first by degree of upfold regulation with 5-aza/TSA treatment and second by COPA upregulation at the 90^th^ percentile. The product of these ranks was used to rank all targets and a significance threshold (α = 0.005) was chosen resulting in 106 genes of which the top 26 genes were evaluated. In order to not exclude genes outside the U133A platform, we also considered all other genes in the U133 Plus 2.0 platform on the sole basis of 5-aza/TSA upfold regulation. Genes were subsequently screened by presence of CpG islands using MethPrimer and all genes were validated by bisulfite sequencing of tumor and normal tissues, and QRT-PCR of cell lines and primary tumors. Of the integrative targets 7/26 passed our validation, while 2/46 of the non-integrative targets passed. Functional experiments were then conducted on these genes. (b) Representative COPA graph of *MAGEA3* demonstrating the statistical approach to finding candidate overexpressed oncogenes. Difference in tumor (n = 49) versus normal (n = 19) expression was significant, p value<0.001 measured by Mann-Whitney U test. (c) Promoter demethylation causes transcriptional upregulation. Upregulation after treatment with 5-aza/TSA is shown in cell lines as measured by QRT-PCR. The ratio of 5-aza/TSA treated expression to baseline is shown for *C19ORF28*, *H19*, *TKLT1*, *GPR17*, *GRIN1*, *MAGEA2*, *MAGEA3/6*, *MAGEA4*, *MAGEA11*. Each gene demonstrated significant upregulation by 5-aza/TSA treatment in at least one cell-line. Error bars show SE.

Concurrently, we performed a comparative epigenetic approach utilizing Cancer Outlier Profiling Analysis (COPA) using 49 primary HNSCC and 19 normal mucosal tissues assayed for mRNA expression on the Affymetrix U133A mRNA expression microarray platform (16,383 probe sets) compiled from prior work and public sources of expression (oncomine.org). COPA is particularly useful to determine differences in expression for particular genes in subsets of primary tumor samples, with improved performance compared to statistical tools that rely on median or average expression difference between two datasets [14]. We calculated COPA at the 90^th^ percentile for our final rankings of all 16,383 features of the arrays, as this resulted in the most pronounced differences in expression with our sample size. Statistical significance of the expression differences in the COPA diagrams were measured by Mann-Whitney U test (Figure 1B).

We determined gene ranks in two ways: 1) COPA ranking at the 90^th^ percentile of upregulation in primary tumor tissue versus normal tissue expression and 2) upfold regulation after pharmacologic demethylation after dChip normalization in cell lines.

An integrative rank product was calculated (Figure 1A). Using a significance threshold (α = 0.005) and subsequent random permutation of our rank-lists, we identified 106 genes that were significantly differentially upregulated based on epigenetic screening and tissue microarray expression (Table S1). We empirically selected the top scoring 26 genes for further analyses. Seventeen of 26 genes containing promoter-associated CpG islands utilizing the MethPrimer software were selected for further studies[15].

In a separate parallel analysis to account for possible activated proto-oncogenes not included in the U133A platform, we analyzed 32,500 genes in the U133plus2 platform ranked on the sole basis of 5-aza/TSA upfold regulation in our normalized cell lines that were not included in primary tumor expression array analysis. We identified 46 target genes with >2-fold upregulation at 90% confidence interval and an average difference value expression over baseline greater than 50. Among these, 30 were confirmed to have CpG islands (Table S2).

### Validation of tumor specific promoter demethylation of target genes

**Figure 2:**
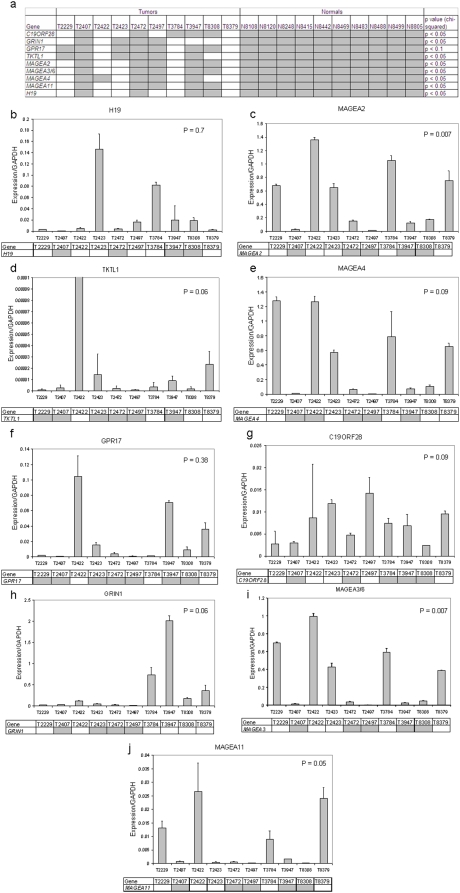
Promoter methylation status in primary tissues. (a) Shown are the bisulfite sequencing results in 10 tumors and 10 normals for: *TKTL1* (4/10, p<0.05), *H19* (6/10, p<0.05), *MAGEA2* (5/10, p<0.05), *MAGEA3/6* (5/10, p<0.05), *MAGEA4* (5/10, p<0.05), *MAGEA11* (5/10, p<0.05), *GPR17* (3/10, p<0.10), *GRIN1* (6/10, p<0.05), *C19ORF28* (5/10, p<0.05). (b–j) QRT-PCR expression with the bisulfite sequencing of the respective promoter below (white is unmethylated, grey is methylated). Significance was measured by comparison of expression of methylated to unmethylated by Mann-Whitney U test. Significance was found in MAGEA2 (p = 0.007), MAGEA3/6 (p = 0.007), MAGEA11 (p = 0.05). Strong associations between expression and promoter methylation status were also found for TKTL1 (p = 0.06), MAGEA4 (p = 0.09), C19ORF28 (p = 0.09), GRIN1 (p = 0.06). H19 (p = 0.7) and GPR17 (p = 0.38) did not show associations between bisulfite sequencing and expression in this cohort. Error bars depict standard error.

CpG islands in the promoter region of the 47 selected gene targets with CpG islands were bisulfite sequenced in normal mucosal samples from patients without a cancer diagnosis to confirm epigenetic silencing in mature upper aerodigestive tract mucosa (Tables S1 & S2). Only 18/47 promoter regions demonstrated complete methylation at all sequenced CpG sites in all normal tissues. These targets were subsequently bisulfite sequenced in 10 primary HNSCC to assay for the presence of hypomethylation. (Figure 2A). Of these targets, 9/18 showed demethylation (see Table 1) in tumor tissues in greater than 30% of the samples, including *TKTL1* (4/10, p<0.05), *H19* (6/10, p<0.05), *MAGEA2* (5/10, p<0.05), *MAGEA3/6* (5/10, p<0.05), *MAGEA4* (5/10, p<0.05), *MAGEA11* (5/10, p<0.05), *GPR17* (3/10, p<0.10), *GRIN1* (6/10, p<0.05), *C19ORF28* (5/10, p<0.05), (chi-squared). To confirm transcriptional upregulation of target genes with 5-aza/TSA treatment in our cell line system (seen in Figure S1), we performed quantitative RT-PCR on 5-aza/TSA-treated normal cells compared to mock-treated cells for these nine genes (Figure 1C). Each gene demonstrated significant upregulation by 5-aza/TSA treatment in at least one cell line supporting functional gene regulation by promoter hypomethylation. Using the initial cohort of 10 primary tumors, we performed a preliminary analysis to determine the relationship of promoter hypomethylation to expression. QRT-PCR expression with the bisulfite sequencing of the respective promoter below is shown in Figure 2b–j. We employed the Mann-Whitney U test to compare QRT-PCR expression of the methylated and unmethylated groups. Three genes had statistically significant increased expression in the unmethylated group: *MAGEA2* (p = 0.007), *MAGEA3/6* (p = 0.007), *MAGEA11* (p = 0.05). Possible associations between expression and promoter methylation status in this small cohort were also suggested for *TKTL1* (p = 0.06), *MAGEA4* (p = 0.09), *C19ORF28* (p = 0.09), *GRIN1* (p = 0.06), yet *H19* (p = 0.7) but *GPR17* (p = 0.38) did not show this association.

**Table 1:** 

Accession	Symbol	Description	COPA Score (Tumor 90th COPA percentile/Normal 90th)	Upregulated with 5-Aza (fold change)	Methylated in Normal Upper Aerodigestive Tissue	Unmethylated in HNSCC Tumor Tissue
NM_021731	C19ORF28	Chromosome 19 ORF 28	N/A	3.7	Y	Y
AL575306	H19	H19 Maternally imprinted	N/A	9.0	Y	Y
Z49258	TKL1	TKL1-transketolase-like	2.5	189.0	Y	Y
NM_005356	GPR17	G protein-coupled receptor 17	15.9	2.8	Y	Y
NM_007327	GRIN1	GRIN1-NMDA receptor 1 isoform NR1-3 precursor	5.1	16.4	Y	Y
U82671	MAGE A2	Melanoma Antigen Family A2	160.0	2.0	Y	Y
BC000340	MAGE A3	Melanoma Antigen Family A3	57.6	2.9	Y	Y
AW438674	MAGE A4	Melanoma Antigen Family A4	39.6	18.0	Y	Y
BC003408	MAGE A11	Melanoma Antigen Family A11	19.8	2.7	Y	Y

### Functional validation of candidate genes

**Figure 3:**
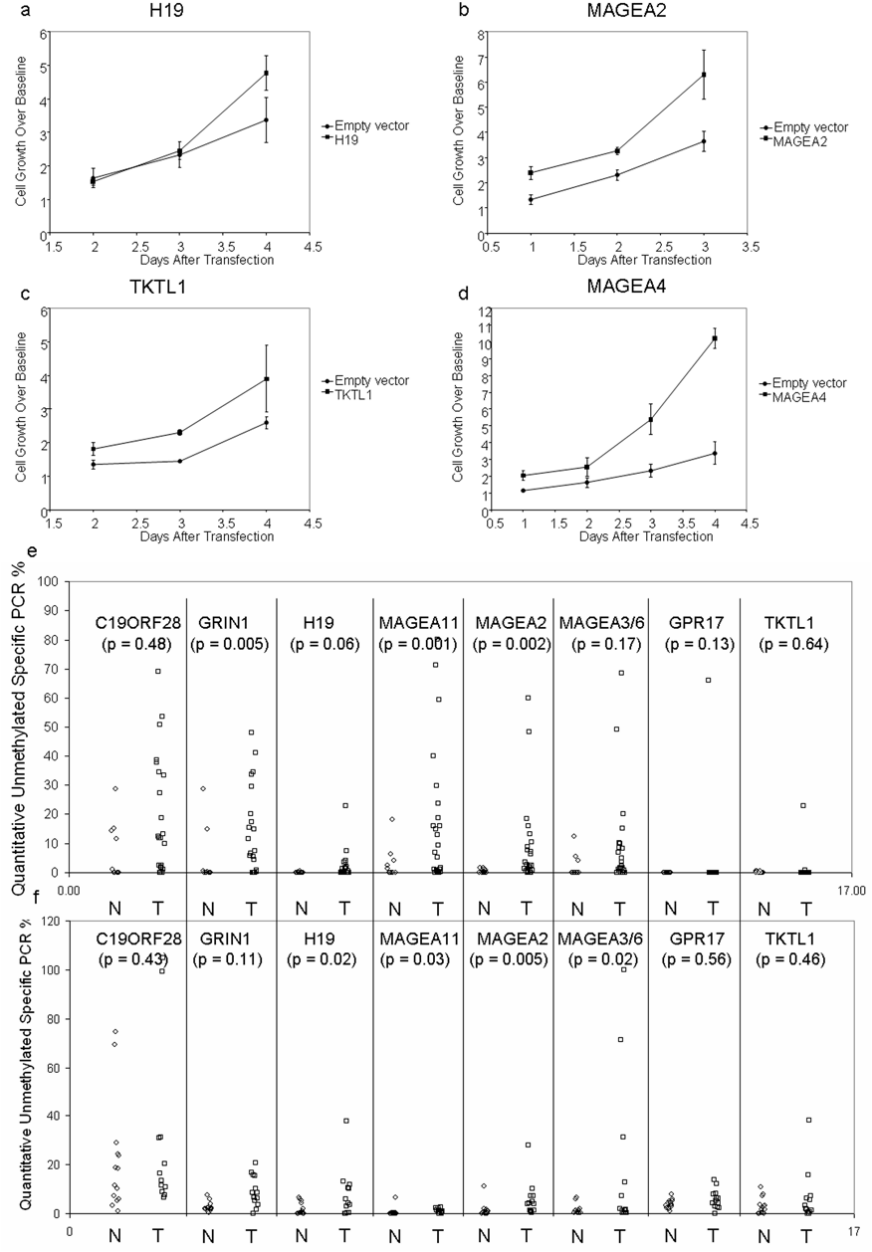
Transient transfection of target genes in minimally transformed oral keratinocytes. (a) Transient transfection of an *H19* construct into OKF6-Tert-1R cells (at day 4, 41.4%±15% growth increase). (b) Transient transfection of an *MAGEA2* construct into OKF6-Tert-1R cells (at day 3, 72.7%±26% growth increase). (c) Transient transfection of an *TKTL1* construct into OKF6-Tert-1R cells(at day 4, 50.1%±38% growth increase). (d) Transient transfection of *MAGEA4* construct into OKF6-Tert-1R cells (at day 4, 203%±17% growth increase). For (e) we developed a quantitative assay for measuring unmethylated promoters, termed Quantitative Unmethylation-Specific PCR (QUMSP). QUMSP percentage of *C19ORF28*, *GRIN1*, *H19*, *MAGEA11*, *MAGEA2*, *MAGEA3/6*, *GPR17*, and *TKTL1* was conducted in a separate cohort of head and neck cancer patients using 25 tumors and 11 upper aerodigestive mucosal samples to assay promoter demethylation. Statistically significant differences were found in *GRIN1*, *MAGEA11*, *MAGEA2*. Next promoter demethylation was considered as a cause of mRNA expression increases seen in the expO dataset. (f) shows the QUMSP results for an independent cohort of 14 lung normals and 13 lung tumor patients. Significant differences in QUMSP were found in *H19*, *MAGEA11*, *MAGEA2*, and *MAGEA3/6*.

We then performed transient transfections to evaluate and/or confirm growth-promoting effects of these nine targets that show tumor-specific promoter hypomethylation. Although H19 codes for a nontranslated RNA transcript, the H19 product appears to induce growth in lung and breast cancer cell lines [16] and may induce drug resistance in hepato-cellular carcinoma [17]. Figure 3A shows results obtained by transient transfection of an *H19* construct into OKF6-Tert-1R cells. At four days, there was a 41.4% (±15%) increase in growth over the transfected empty vector. The MAGE family consists of related family members that are known to be upregulated in a variety of tumor types[18], but have recently been implicated in inducing transcriptional reprogramming in tumor cells[19]. *MAGEA2* induced a 72.7% (±26%) increase in growth at day three (Figure 3B). *MAGEA4* transfection induced a 203% (±17%) increase in growth (Figure 3D). Functional growth differences were tested, but not found for *C19ORF28*.

**Figure 4:**
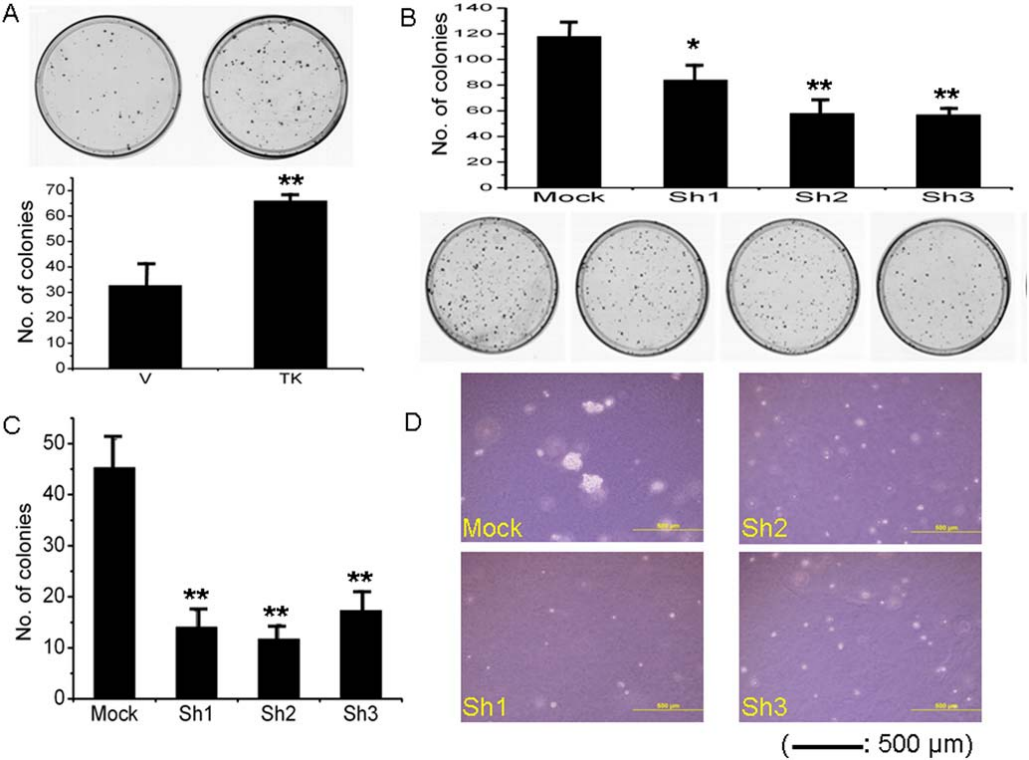
TKTL1 transient transfection and transcriptional repression with shRNA. (a) TKTL1 forced overexpression via transient transfection in background low expressing JHU-011 cells induces increased anchorage dependent colony formation and (b) TKTL1 shRNA in high-expressing FaDu cell line induces growth inhibition. (c) Anchorage independent growth of UM-22B cells is significantly inhibited by TKTL1 shRNA (d), with decrease in colony size. (* = p<0.01, ** = p<0.001, chi square).

In Figure 3C, *TKTL1* induced a 50.1% (±38%) increase in growth at day four. Enhanced expression of TKTL1 has recently been implicated in the conversion of cells to aerobic, glycolytic metabolism as well as increased proliferation in colon cancer cells [20]–[25]. TKTL1 is independently associated with poor survival in laryngeal carcinoma, colon and urothelial cancers, as well as distant metastasis in ovarian carcinoma [22], [23], [26] To further confirm TKTL1 as a candidate proto-oncogene in HNSCC, we performed adherent colony focus assays in TKTL1 low- expressing HNSCC cell lines JHU-011 and JHU-028, and found significant growth increase in both cell lines (Figure 4 A,B). We then employed shRNA constructs in a TKTL1 high-expressing cell line UM-22B in anchorage independent growth assays, and noted a dramatic decrease in size and number of colonies (Figure 4 C,D) compared to mock transfected cells.

### Candidate proto-oncogene expression and promoter demethylation in other human cancer types

**Figure 5:**
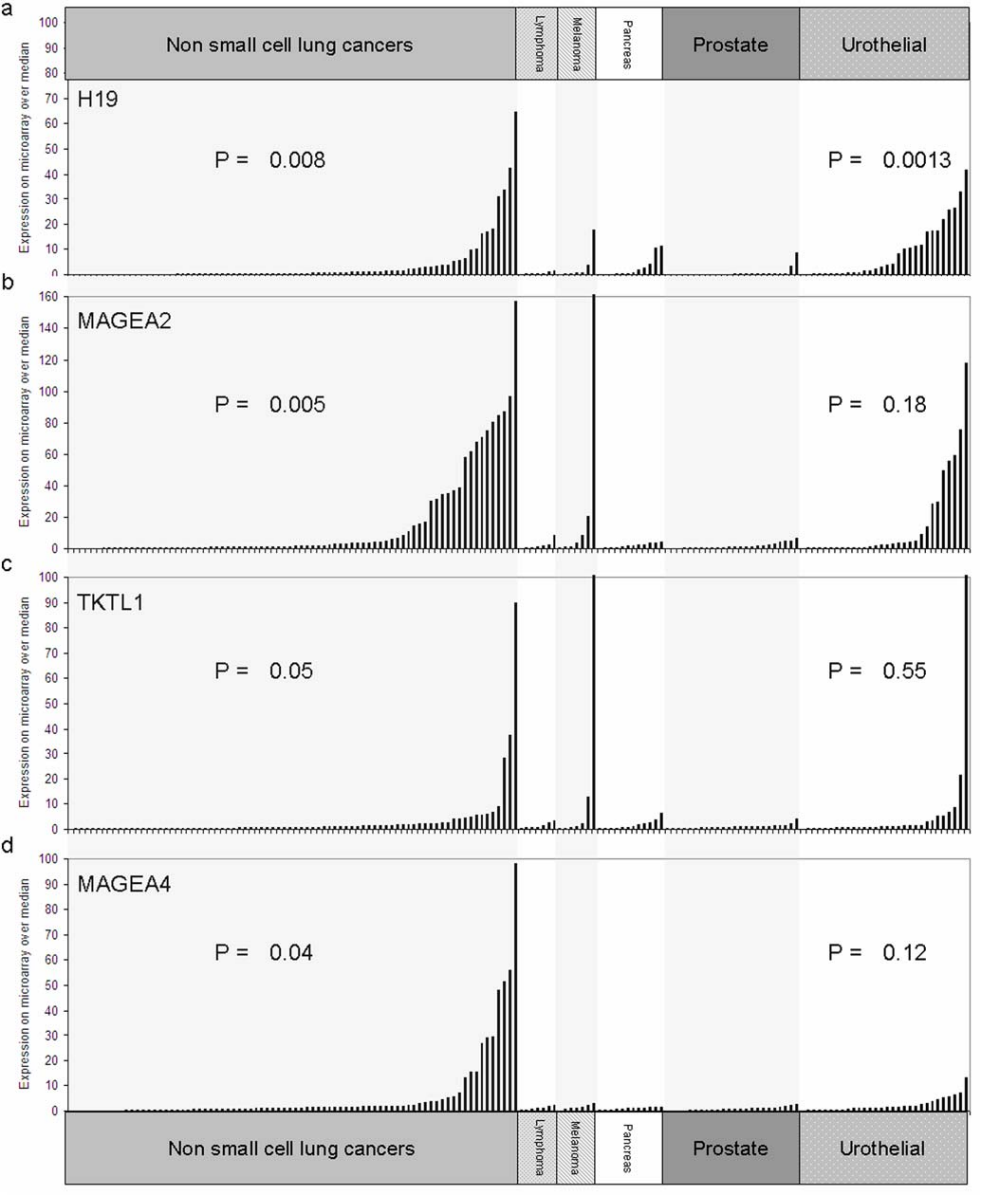
Overexpression and demethylation in other human cancers. The expO dataset repository was mined for tumor tissue gene expression measured by the Affymetrix U133 Plus 2.0 mRNA expression platform. Initially data was median-normalized by expression array and each gene was median normalized for this figure. Only the subsets of non small cell lung cancer, lymphoma, melanoma, pancreas cancer, and urothelial cancer are displayed. (a) shows the expression of *H19* in these cancers. (b) *MAGEA2* expression, (c) *TKTL1* expression, and (d) *MAGEA4*. Statistical significance was measured in each tumor type by comparing gene expression in the tumor type to expression in all the remaining 1041 samples. Tumor types without p values did not approach statistical significance. Lung and urothelial showed significant expression overlap.

To determine if candidate proto-oncogene expression was altered in a broader range of tumor types, we analyzed expression data available through the expO datasets for 1041 human tumors of all histologies [27]. Data was first median-expression normalized by each array and subsequently by median normalization by probe set feature across the 1041 tumors from many cancer types including lung and urothelial, but not HNSCC. We chose a subset of these tumors, non-small cell lung cancer (NSCLC), lymphoma, melanoma, pancreatic cancer, prostate cancers, and urothelial cancers, for presentation (Figure 5A–D). *H19* was significantly upregulated in NSCLC (p = 0.008) and in urothelial cancer (p = 0.0013), as calculated by Mann-Whitney U test comparing array-normalized expression in tumor type to all other tumors. We noted significantly increased expression of MAGEA2 in NSCLC (p = 0.005) but not in urothelial cancers (p = 0.18). *TKTL1* also showed overexpression in NSCLC (p = 0.05), but not urothelial cancer (p = 0.55), and *MAGEA4* was overexpressed in NSCLC (p = 0.04), but not significantly so in urothelial cancer (p = 0.12). In order to confirm target-specific demethylation noted in primary tumors, we devised a rapid, quantitative assay for specifically measuring non-methylated promoters, which we termed Quantitative Unmethylation-Specific PCR (QUMSP). Twenty-five HNSCC tumors and 11 upper aerodigestive mucosal samples were assayed for promoter demethylation (Figure 3E). Tumor-specific demethylation was found in *GRIN1* (p = 0.005), *MAGEA11* (p = 0.001), and *MAGEA2* (p = 0.002). We performed a similar analysis using a separate, independent cohort of 13 NSCLC samples with 14 lung samples from patients without neoplastic disease and confirmed promoter hypomethylation in target genes. Significant differences at 〈<0.05 in QUMSP were found in *H19* (p = 0.02), *MAGEA11* (p = 0.03), *MAGEA2* (p = 0.005), and *MAGEA3/6* (p = 0.02). See Figure 3F.

### Aberrant expression of candidate proto-oncogenes occurs in a coordinated fashion in individual primary tumors

**Figure 6:**
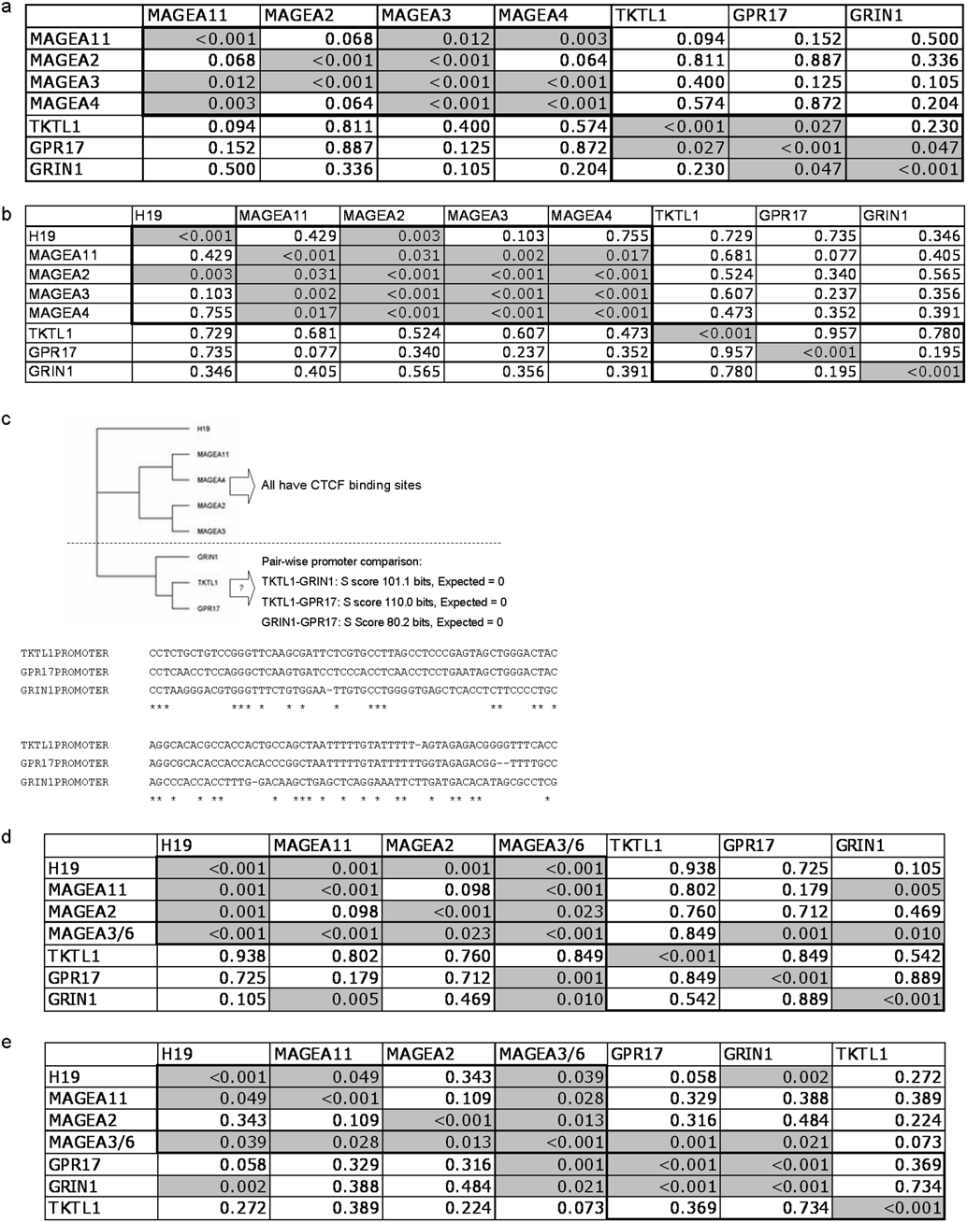
Gene expression and demethylation correlation. (a) Shows the gene expression correlation p-value matrix for the coexpression for each gene pair across all tumors. This comparison shows the correlation of each gene pair in 49 head and neck tumors. (b) Gene pair expression p-value correlation matrix for 80 NSCLC. Of note C19ORF28 is not tiled on this array platform. (c) Analysis of promoter regions for the genes. Shown is a phylogram of our promoters of interest based on ClustalW analysis after multiple sequence alignment. The region of significant homology is shown after sequence alignment and E statistics from EMBL-EBI's PromoterWise comparison. (d) Promoter hypomethylation (QUMSP) correlation p-value matrix for HNSCC (25 tumors). (e) Promoter hypomethylation (QUMSP) correlation p-value matrix for NSCLC (13 tumors).

During these analyses, we quickly noted that transcriptional upregulation via promoter hypomethylation tended to occur synchronously in a subset of tumors. In our cohort of 49 primary HNSCC assayed via expression array analysis, we constructed a matrix of Pearson's correlation coefficients between the expression levels of each target (Figure 6A). For our nine target genes, significant clustering of increased expression was noted within the MAGEA family of genes. H19 was not included because of its absence on the U133A platform. A separate cluster of associated overexpression was noted for *TKTL1*, *GRIN1*, and *GPR17*. From NSCLC expression data derived from the expO datasets we created similar matrices to examine correlations between individual genes. We noted that MAGEA family expression and *H19* expression showed highly significant correlations in individual NSCLC (see Figure 6B). In contrast, there were no target-target correlations for NSCLC expression of the other cluster (*TKTL1*, *GRIN1*, and *GPR17*) that exhibited coordinated expression in HNSCC.

### Expression patterns correlate with promoter homology for promoter demethylated target genes

We then wanted to determine if promoter homology was associated with the linked expression of the two proto-oncogene clusters. We subsequently used the European Bioinformatics Institute's ClustalW tool (Figure 6C) for phylogram analysis after multiple sequence alignment of the respective promoters. To confirm homology quantitatively, we used EMBL-EBI's PromoterWise comparison tool which found significant pair-wise areas of promoter homology in *GPR17*, *GRIN1*, and *TKTL1*. As expected from earlier studies, the MAGE-A family clustered together, as the MAGE-A family members and H19 are known to have consensus-binding sites for methylation-sensitive binding factors *CTCF* and *CTCFL*/*BORIS*. In addition, this second group of *GRIN1*, *GPR17*, and *TKTL1* clustered together by sequence homology.

Finally, we wanted to see if the degree of promoter hypomethylation was correlated in individual tumors. For both primary HNSCC (Figure 6D) and NSCLC (Figure 6E), multiple significant correlations between methylation status were found between targets, but methylation status did not cluster in groups defined by the MAGE-A family/H19 expression cluster or by the *TKTL1*, *GRIN1*, *GPR17* cluster. Rather, there were significant correlations between all identified candidate proto-oncogenes. Hypomethylation, therefore, appeared to occur in a related fashion in individual tumors for all target genes, but the concurrent expression of genes within the two clusters was associated with promoter homology rather than methylation status. This implied that specific transcriptional factors may be involved in the regulation of epigenetic unmasking and/or transcriptional activation based on promoter homology among these candidate proto-oncogenes.

### BORIS expression is associated with proto-oncogene activation in primary tumors, induces promoter demethylation, candidate proto-oncogene expression, and cell transformation

**Figure 7:**
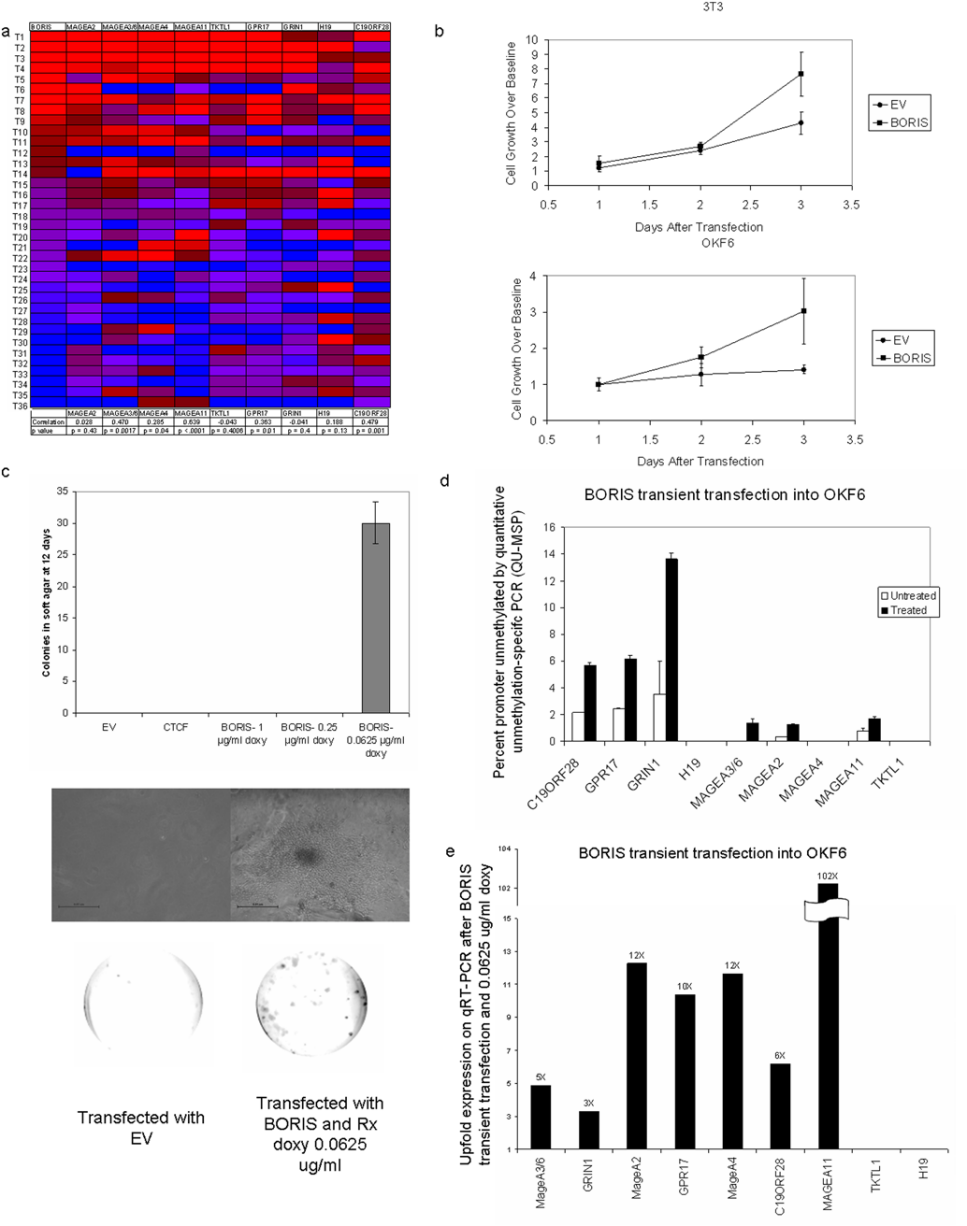
BORIS transfection studies. (a) *BORIS* expression correlates with expression of target genes in HNSCC (QRT-PR) heat map (Pearson correlation) (b) Transient transfection of *BORIS* construct into NIH-3T3 and OKF6-Tert1R cell lines. *BORIS* overexpression resulted in increased cell growth in the 3T3 cell line (at day 3, 77%±34% growth increase) and in the OKF6-Tert1R cell line (at day 3, 161%±78% growth increase). Cell growth over baseline calculated by dividing values by day 1's Calcein signal. (c) Anchorage independent growth was assayed after transfection with empty vector (EV), CTCF, and BORIS at various concentrations of doxycycline, with representative colony (below). (d) QUMSP of nine targets of interest after transfection with empty vector (untreated) and BORIS construct (treated) in presence of 0.0625 µg/mL of doxycycline. (e) Fold increase Quantitiative RT-PCR of nine targets of interest after BORIS transfection normalized to values after transfection with empty vector.

The obvious presence of several MAGE genes among our targets prompted us to study upstream regulatory pathways of known cancer-testis antigens. BORIS and CTCF are a unique cognate pair of transcriptional factors involved in epigenetic regulation that share an identical DNA-binding domain. BORIS is transcriptionally silenced in most normal tissues, but expressed in normal embryonic, germ cell, and cancer tissues. We determined if expression of BORIS correlated with candidate proto-oncogene expression in a separate cohort of 36 primary HNSCC. Figure 7A presents a heat map constructed from median normalized, qRT-PCR expression data of our proto-oncogenes, sorted by BORIS expression. In these 36 cancers, BORIS overexpression was significantly correlated to overexpression of 6/9 proto-oncogenes including: *MAGEA3/6* (p = 0.0017), *MAGEA4* (p = 0.04), *MAGEA11* (p<0.001), *GPR17* (p = 0.01), and *C19ORF28* (p = 0.001). To further examine the correlation of BORIS expression with our target genes in solid cancers, we analyzed the expO dataset data for 1041 human tumors of a wide variety of tissue sources and histologies. Significant positive correlation of BORIS expression with expression of each of our nine proto-oncogenes was noted: *GRIN1* (p<0.001), *C19ORF28* (p<0.001), *H19* (p<0.001), *MAGEA11* (p<0.001), *MAGEA2* (p<0.001), *MAGEA3/6* (p = 0.003), *MAGE4* (p<0.001), *TKTL1* (p<0.001), *GPR17* (p<0.001), (Figure S2). Although BORIS transcripts are usually undetectable in normal cells, we determined that 59% of all tumors have a BORIS level that exceeds the median expression of all genes, and 90% of tumors have a BORIS expression level >25% of median expression value for all genes, indicating that aberrant BORIS expression is a common event in human cancer.

To explore the functional and epigenetic effects of BORIS, tetracycline inducible pBIG2i-BORIS constructs were transiently transfected into NIH-3T3 and OKF6-Tert1R cell lines in the presence of doxycycline, resulting in increased adherent cell growth in wild type, BORIS non-expressing NIH3T3, and OKF6-Tert1R cell lines. 3T3 cells had a 77%±34% growth increase at day three. OKF6 cell lines had a 161%±78% growth increase at day three (Figure 7B). Importantly, these effects were seen when levels of *BORIS* expression was regulated to be similar to the levels found in primary tumors.

This effect was not seen with increased concentrations of doxycycline that induced high levels of *BORIS* transcripts. An analysis of transcripts showed that expression of seven of nine target genes was significantly increased in OKF6-Tert1R cell expressing BORIS (Figure 7E). To test if BORIS expressed at low levels might contribute to transformation, we studied NIH3T3 cells for anchorage independent growth. After 12 days, significant numbers of colonies (30+/−3) were observed in tests of BORIS-expressing cells but not in cells transfected with a control plasmid (Figure 7C).

Finally, to test the possibility that BORIS may be associated with epigenetic alterations as well as transcriptional upregulation of our target genes, we quantitatively assayed for methylation status of our candidate proto-oncogenes after BORIS transfection and noted that six out of nine targets (C19 ORF28, GPR17, GRIN1, MAGEA2, MAGEA3/6, and MAGE11) showed a greater than 100% increase in demethylated promoter as early as 48 hours after induction of BORIS (Figure 7D).

## Discussion

The data presented above indicate that HNSCC and NSCLC undergo activation of candidate proto-oncogenes with associated demethylation in a coordinated fashion in individual tumors. We were able to demonstrate transformation-associated effects of BORIS expressed ectopically in BORIS-negative cell lines as well as growth effects with individual target genes that have been shown to be epigenetically activated and expressed by BORIS. However, this does not rule out the contribution of as yet unidentified genes to BORIS related effects or a cooperative effect between identified target genes. The lack of direct correlation of demethylation of promoter and increased expression in some of these targets (Figure 2) may simply be a reflection of the small cohort used to test this association in HNSCC, but may also be due to alternate mechanisms of transcriptional control of these genes other than promoter methylation status, including other promoters, repressors, or mediators (e.g. BORIS). Cancer testes antigens include four of our genes, *MAGE A2*, *A3/6*, *A4*, *A11*, are part of the melanoma antigen family A (MAGE-A) family of genes initially discovered as targets for immunotherapy due to their near exclusive tumor-specific expression, but the MAGE-A family plays a functional role in cancer development [28]. MAGEA2 binds to p53-responsive promoters and leads to assembly of a p53/MAGEA2/HDAC3 protein complex, resulting in transcriptional silencing of genes ordinarily activated by p53 because of histone deacetylation. Additionally, different MAGE-A family members can repress downstream targets of p53, and studies have also linked MAGE-A family overexpression to chemo-resistance[29], [30], and MAGE family members have been shown to increase cell growth and inactivate TSG activity [31], [32]. Recently, MAGEA has been shown to repress p53-dependent apoptosis, and has been associated with resistance to taxanes and alkylating agents in gastric cancer [33], [34]. We found these MAGE family members to show significant expression in a correlated fashion in HNSCC and NSCLC, and to reexpress in cell lines treated with 5-aza-deoxycytidine. In primary tissue some targets expression level was directly correlated to promoter methylation status.

We found that expression of the MAGE-A family and expression of *H19* appeared to be significantly related in our primary tumors, supported by data indicating that these targets are controlled by common methylation-specific transcription factors [35], [36]. *H19* forms half of the best-studied example of imprinted-gene regulation, the *IGF2/H19* locus. *IGF2* (insulin-like growth factor 2) is expressed uniquely from the parental allele achieved by monoallelic methylation of the imprinting control region (ICR) at 11p15.5 [37]. Aberrant hypomethylation at this locus is one cause of Silver-Russell syndrome—a disease of asymmetry or hemihypertrophy associated with increased risk of malignancies including craniopharyngioma, testicular seminoma, hepatocellular carcinoma, and Wilms tumor [38]. Additionally, several cases of familial Beckwith-Wiedemann syndrome (BWS), with and without Wilms' tumors, have been shown to be caused by microdeletions of the methylation-specific CTCF binding sites in the *H19* ICR, a rare familial cancer syndrome linked to epigenetics [39]–[41]. H19 and the MAGE family members showed significant correlation in this expression and demethylated promoter status in HNSCC and NSCLC.

Other identified proto-oncogenes in this report have been implicated recently in tumorigenesis. *TKTL1* protein expression is correlated to worse outcome in patients with invasive colon and urothelial tumors, and investigators hypothesize that enhanced *TKTL1* expression in tumors increases oxygen-independent glucose usage [22]. In addition, over-expression of TKTL1 has since been validated as a potential biomarker and treatment target in breast cancer [20]. *GPR17* and *GRIN1* have not been implicated in carcinogenesis to date. Although we were unable to demonstrate growth promoting effects of *C19ORF28*, this does not exclude the possibility that overexpression of this and any of our other targets may contribute to a malignant phenotype in other cell backgrounds, or via other mechanisms—i.e., motility, invasion, angiogenesis, or apoptosis resistance—or that it may cooperate with other identified targets to produce phenotypic effects.

The epigenetic reactivation of *TKTL1*, *H19*, *MAGEA2*, *MAGEA3/6*, *MAGEA4*, *MAGEA11*, *GPR17*, *GRIN1*, and *C19ORF28*, genes located at diverse chromosomal loci, occurs simultaneously in individual primary tumors from multiple tumor types. This concurrent genome-wide, promoter-specific hypomethylation that results in derepression of many potential oncogenes raises the possibility of a demethylator phenotype analogous to the CpG island methylator phenotype (CIMP) initially noted in colon cancer [42], [43]. Many proto-oncogenes are members of the cancer testes antigen family which are ordinarily repressed via epigenetic mechanisms during development. An attractive hypothesis is that this phenomenon represents the coordinated, but pathologic reversal of developmental epigenetic regulatory patterns in cancer cells. The validity of our whole-genome integrative approach to screening for epigenetically-activated genes associated with malignancy is, in fact, confirmed by the appearance of *H19* and the MAGE-A family members which have been reported to be controlled by epigenetic activation and show silencing in normal cells. Two separate groups among our nine genes showed statistically significant correlations for patterns of expression: 1) MAGEA family members with *H19* and 2) *TKTL1*, *GPR17*, and *GRIN*. We were also able to define these groups according to promoter homology, implicating the participation of promoter-specific binding activity in the coordinated expression of each of these groups and suggesting the existence of additional common transcriptional activators that recognize the specific demethylated promoter sequences of these genes. The strict correlation of BORIS expression with aberrant expression of multiple growth-promoting proto-oncogenes in a variety of solid tumors reinforces the postulated role for BORIS as a key participant in aberrant demethylation and transcriptional activation of putative oncogenes. This concept is supported by cell line experiments demonstrating that BORIS expression by itself is sufficient to simultaneously demethylate and activate the transcription of these genes. However, some reports have shown melanoma tissue samples that express MAGE-A1 in the absence of BORIS activation, suggesting that BORIS is not an obligate factor for activation of these genes [44]. It is of great interest to define the factors with which BORIS cooperates to induce these epigenetic and expression changes. Recently, a role for BORIS in histone demethylation and chromatin remodeling has been demonstrated [45]. Moreover, regardless of mechanism, our data provide strong evidence for consideration of BORIS as a dominant controlling factor for facilitating epigenetic alterations associated with coordinated demethylation and reactivation of target genes that are of high value as potential therapeutic and diagnostic targets for NSCLC, HNSCC, and other tumors.

This simultaneous reactivation of multiple targets provides a significant challenge to the understanding of the collective, and perhaps cooperative, effects of this phenomenon in cell transformation. In particular, single targets may depend on concurrent activation of, and interaction with, other family members for oncogenic effect. Other investigators have found some evidence of coordination of cancer testes antigen family expression and the possibility of direct interactions [46], [47]. In addition, we selected only the top 26/106 possible targets identified after integrative analysis in a single solid tumor type for further analysis. We would expect that future studies of the remaining genes, as well as use of normal cell lines and tumors derived from other tissues in an integrative approach, will allow for discovery of additional, novel, epigenetically-controlled genes that may also act collaboratively to induce malignant transformation.

Due to lack of primary tumor data on a larger array platform we also used a nonintegrative approach, which resulted in ultimate validation of 4.3% of the targets (2/46) compared to the integrative results that produced a 27% hit rate (7/26), reflecting a higher ability to validate targets in primary tumor when these data are included in initial discovery strategies. Additional analysis of other targets that are significantly differentially regulated may also yield additional epigenetically derepressed targets. Finally, these data have therapeutic implications for demethylation therapy and targeting of therapy. The active investigation of pharmacologic demethylating agents as therapy for malignancy based on reversal of silencing of tumor suppressor genes may have unintended effects. It is possible that in certain tissues this may result in reactivation of developmentally repressed proto-oncogene targets, with the unintended effect of promoting late, second primary tumors [48]. However, modulation of a pathway that involves the coordinated derepression of a series of growth-promoting proto-oncogene candidates and a key transcriptional effector, BORIS, may provide a significant opportunity for directed therapeutic intervention that simultaneously targets multiple oncogenic pathways.

## Materials and Methods

### Histopathology

All samples were analyzed by the Pathology department at Johns Hopkins Hospital. Tissues were obtained via Johns Hopkins Institutional Review Board approved protocols under JHM IRB Protocol #92-07-21-01, “Detection of Genetic Alterations in Head and Neck Tumors.” Normal samples were microdissected and DNA prepared from the mucosa. Tumor samples were confirmed to be head and neck squamous cell carcinoma and subsequently microdissected to separate tumor from stromal elements to yield at least 80% tumor cells. Tissue DNA was extracted as described below.

### 5Aza-dC and TSA Treatment of Cells

These *in vitro* techniques employ treatment of cultured cells with 5-aza-deoxycytidine (a cytosine analog which cannot be methylated) with or without Trichostatin A (a histone deacetylase inhibitor) and subsequent expression array analysis with validation of tumor suppressor gene targets [13]. We treated HNSCC cell lines with 5Aza-dC and/or TSA as described previously. Briefly, cells were split to low density (1×10^6^ cells/T-75 flask) 24 hours before treatment. Stock solutions of 5Aza-dC (Sigma, St. Louis, MO) and TSA (Sigma) were dissolved in DMSO (Sigma) and 100% ethanol, respectively. Cells were treated with 5 µM 5-Aza-deoxycytidine for 5 days and 300 nM TSA for last 24 hours. Baseline expression was established by mock-treated cells with the same volume of DMSO or ethanol. Two normal oral keratinocyte cell lines (OKF6-Tert1 and OKF6-Tert1R, immortalized with hTert, a generous gift from J. Rheinwald, Harvard), were treated in duplicate by 5-azadeoxycytidine/trichostatin A.

### Oligonucleotide microarray analysis and QRT-PCR analysis

Total cellular RNA was isolated using the RNeasy kit (Qiagen, Valencia, CA) according to the manufacturer's instructions. We carried out oligonucleotide microarray analysis using the GeneChip U133plus2 Affymetrix expression microarray (Affymetrix, Santa Clara, CA). Samples were converted to labeled, fragmented, cRNA per the Affymetrix protocol for use on the expression microarray. Signal intensity and statistical significance was established for each transcript using dChip version 2005. Two-fold increase based on the 90% confidence interval of the result and expression minus baseline >50 was used as the statistical cutoff value after 5Aza-dC and/or TSA treatment to identify upregulated candidate genes.

### Public datasets

The public databases used in this study were the University of California Santa Cruz (UCSC) Human Genome reference sequence and the annotation database from the May 2004 freeze (hg17). Fifty-six HNSCC expression microarrays were obtained from public datasets from Oncomine (Oncomine.org, Ann Arbor, Michigan). Fourteen expression microarrays that our laboratory had previously studied from the same platform were incorporated and all microarrays were normalized for COPA analysis. We also utilized the expO datasets (1185 tumors on the Affymetrix U133plus2 mRNA expression platform) available online as part of the Gene Expression Omnibus (GEO/NCBI) and kindly provided by the International Genomics Consortium. This data is publicly available online as part of the Gene Expression Omnibus (GEO/NCBI), produced by the International Genomics Consortium. This analysis utilized expression array data for 47,000+ genes measured in 1041 human tumors of various histologies.

### Cancer outlier profile analysis (COPA)

Heterogeneous patterns of proto-oncogene activation have been noted, and traditional approaches such as determining average fold differences, t-tests, and other techniques may fail to define significant alterations in expression for specific genes in high-throughput array approaches [14]. We applied COPA to our cohort of 68 tissues (49 tumors, 19 normals), with each gene expression data set containing 14,500 probe sets. Briefly, gene expression values are median centered, setting each gene's median expression value to zero. The median absolute deviation (MAD) is calculated and scaled to 1 by dividing each gene expression value by its MAD. Of note, median and MAD were used for transformation as opposed to mean and standard deviation so that outlier expression values do not unduly influence the distribution estimates, and are thus preserved post-normalization. Finally, the 75^th^, 90^th^, and 95^th^ percentiles of the transformed expression values are calculated for each gene and then genes are rank-ordered by their percentile scores, providing a prioritized list of outlier profiles. For the purposes of our rank-list, the 90^th^ percentile was chosen based on sample-size analysis (49 tumors, 19 normals). For details of the method refer to Tomlins et. al.[14].

### Integrative epigenetics

We ranked target genes from the Affymetrix U133A mRNA expression microarray platform by COPA upregulation at the 90^th^ percentile (from 49 tumors and 19 normal tissues). The U133A microarray platform (Affymetrix, Santa Clara California) has approximately 14,500 probe sets. A second rank list was produced by ranking genes in descending order of the degree of upfold regulation upon 5-aza/TSA treatment. These two sources of information (gene set demonstrating upregulation with 5-aza) and COPA score were combined by using a rank product. These two rankings were combined to rank all targets and permutation of the data was used to establish significance with a threshold of 〈 = 0.005. This resulted in 106 genes deemed significant. The top 26 of these targets were comprehensively evaluated. Presence of CpG islands in these genes was determined by MethPrimer. In order to not exclude genes outside the U133A platform, we also considered all other genes in the U133plus2 platform on the sole basis of 5-aza/TSA upfold regulation. For all genes which did not have tissue mRNA expression array information amenable to COPA analysis, we considered only statistically significant reexpression after 5-aza treatment. 46 genes were studied that had an experimental versus baseline expression (E/B) >2.0, based on the 90% confidence interval and E-B >50. All genes were then studied for the presence of CpG islands in promoters or the first intron. Initially, an *in silico* approach was used to confirm the presence of a CpG island using the UCSC genome browser which relies on GC content of >50%, >200 bp, >0.6 observed to expected CG's.

### DNA extraction

Samples were centrifuged and digested in a solution of detergent (sodium dodecylsulfate) and proteinase K, for removal of proteins bound to the DNA. Samples were first purified and desalted with phenol/chloroform extraction. Digested sample was subjected twice to ethanol precipitation, and subsequently resuspended in 500 µL of LoTE (EDTA 2.5 mM and Tris-HCl 10 mM, p 7.5) and stored at −80°C.

### Bisulfite treatment

DNA from salivary rinses was subjected to bisulfite treatment, as described previously[49]. In short, 2 µg of genomic DNA was denatured in 0.2 M NaOH for 30 minutes at 50°C. This denatured DNA was then diluted into 500 µL of a solution of 10 mM hydroquinone and 3 M sodium bisulfite. This was incubated for 3 hours at 70°C. After the DNA sample was purified with a sepharose column (Wizard DNA Clean-Up System; Promega, Madison, WI). Eluted DNA was treated with 0.3 M of NaOH for 10 minutes at room temperature, and precipitated with ethanol. This bisulfite-modified DNA was subsequently resuspended in 120 µL of LoTE (EDTA 2.5 mM and Tris-HCl 10 mM) and stored at −80°C.

### Bisulfite Sequencing

Bisulfite sequence analysis was performed to check the methylation status in primary tumors and normal tissues, as well as cell lines. Bisulfite-treated DNA was amplified using primers designed by MethPrimer to span areas of CpG islands in the promoter or first intron [15]. Primer sequences were designed to not have CG dinucleotides (see Table S3). Detailed primer sequences and PCR conditions are available upon request. The PCR products were gel-purified using the QIAquick Gel Extraction Kit (Qiagen), according to the manufacturer's instructions. Each amplified DNA sample was applied with nested primers to the Applied Biosystems 3700 DNA analyzer using BD terminator dye (Applied Biosystems, Foster City, CA). Of note, due to significant sequence homology of MAGEA3 and MAGEA6, differential sequencing of these genes could not be performed, data are reported for consensus sequence as MAGEA3/6.

### QUMSP

To selectively amplify demethylated promoter regions in genes of interest, probe and primers were designed using data from bisulfite sequencing of primary tumors which are complementary only to bisulfite-converted sequences known to be demethylated in tumor. Probe and primer combinations were validated using *in vitro* methylated and demethylated controls, sequences are provided online at http://www.hopkinsmedicine.org/headneckcancer/headneckinfo.html.

### qRT-PCR

Total RNA was measured and adjusted to the same amount for each cell line, and then cDNA synthesis was performed using oligo-dT with the SuperScript First- Strand Synthesis kit (Invitrogen). The final cDNA products were used as the templates for subsequent PCR with primers designed specifically for each candidate gene. GAPDH was examined to ensure accurate relative quantitation in QRT-PCR. Detailed PCR conditions and primer sequences are available upon request. QRT-PCR heat maps were generated after median-normalization and log-transformation. Heat maps were generated using an Excel.

### Transfection of human expression vectors

Full-length ORF cDNAs of *MAGEA2B*, *MAGEA4*, *H19*, *TKTL1 in pCMV-SPORT6* were obtained for transient transfections. Cell lines were plated at 2×10^5^/well using 6-well plates and transfected with either empty vector or gene of interest using the FuGene 6 Transfection Reagent (Roche, Basel, Switzerland) according to the manufacturer's protocol. Calcein florescence was measured by the Spectramax M2e 96-well fluorescence plate reader Molecular Devices (Sunnyvale, California). Live cells are distinguished by the presence of ubiquitous intracellular esterase activity, determined by the enzymatic conversion of the virtually nonfluorescent cell-permeable calcein AM to the intensely fluorescent calcein. The polyanionic calcein dye is well retained within live cells, producing an intense uniform green fluorescence (excitation/emission ∼495 nm/515 nm). Transfection efficiency was determined with GFP plasmids and was approximately 50% in OKF6 cells. Transgene expression determined by qRT-PCR. *BORIS* expression plasmid pBIG2i-BORIS was used for BORIS transfections [50].

### Anchorage-independent growth assay

Soft agar assays were conducted after transfection of cells with mammalian expression vectors. Cells were counted and approximately 5000 were added into each 6-well plate. The bottom layer was composed of 0.5% agar, DMEM+10% FBS, plus additives, while the cells were suspended in a top layer of 0.35% agar, DMEM+10% FBS, plus additives. BORIS Inducible promoter constructs were incubated in the presence of low doxycycline (0.01 mg/ml). Soft agar assays were incubated at 37 degrees for 2 weeks.

### Statistical analysis

The QUMSP data was analyzed using a Wilcoxon-Mann-Whitney rank test. The p-values were corrected using the Benjamini-Hochberg procedure [51], and significance was defined as pcorr<0.05. We looked for similarities in the methylation patterns between genes by performing an analysis of correlations between QUMSP readings on the genes across all samples. We used 1000 permutations of the samples to establish significance, with α = 0.05. For the expression data, we log-transformed the normalized data and performed correlation analysis across all samples between each of the genes in the study. Significance was determined by assuming a normal distribution in the log-transformed expression levels and applying Student's t-distribution with an alpha of 0.05. All analyses were performed using Matlab. Comparisons of promoter homology were done with European Bioinformatics Institute's ClustalW sequence alignment and phylogram software and the PromoterWise application. Pearson Product Moment Correlation (Pearson's correlation), reflecting the degree of linear relationship between two variables were calculated with Matlab.

## Supporting Information

Figure S1 Upfold regulation of mRNA expression in treated minimally-transformed cell lines measured by Affymetrix U133 Plus 2.0.
(0.05 MB TIF)

Figure S2 BORIS correlates with gene expression in all cancers (using the expO cohort of 1041 human cancers of various tumor sites and histologies). Shown are microarray median-normalized expression of our targets compared to BORIS expression in 1041 human cancers.
(0.03 MB TIF)

Table S1 106 genes differentially upregulated based on epigenetic screening and tissue microarray expression.
(0.04 MB XLS)

Table S2 Target genes ranked on 5-aza/TSA upfold regulation in our normalized cell lines.
(0.02 MB XLS)

Table S3 Primer sequences.
(0.02 MB XLS)

## References

[1] Das PM, Singal R (2004). DNA methylation and cancer.. J Clin Oncol.

[2] Dunn BK (2003). Hypomethylation: one side of a larger picture.. Ann N Y Acad Sci.

[3] Ehrlich M (2002). DNA methylation in cancer: too much, but also too little.. Oncogene.

[4] Gaudet F, Hodgson JG, Eden A, Jackson-Grusby L, Dausman J (2003). Induction of tumors in mice by genomic hypomethylation.. Science.

[5] Nishigaki M, Aoyagi K, Danjoh I, Fukaya M, Yanagihara K (2005). Discovery of aberrant expression of R-RAS by cancer-linked DNA hypomethylation in gastric cancer using microarrays.. Cancer Res.

[6] Zhou H, Chen WD, Qin X, Lee K, Liu L (2001). MMTV promoter hypomethylation is linked to spontaneous and MNU associated c-neu expression and mammary carcinogenesis in MMTV c-neu transgenic mice.. Oncogene.

[7] Watt PM, Kumar R, Kees UR (2000). Promoter demethylation accompanies reactivation of the HOX11 proto-oncogene in leukemia.. Genes Chromosomes Cancer.

[8] Hanada M, Delia D, Aiello A, Stadtmauer E, Reed JC (1993). bcl-2 gene hypomethylation and high-level expression in B-cell chronic lymphocytic leukemia.. Blood.

[9] Mangues R, Schwartz S, Seidman I, Pellicer A (1995). Promoter demethylation in MMTV/N-rasN transgenic mice required for transgene expression and tumorigenesis.. Mol Carcinog.

[10] Feinberg AP, Vogelstein B (1983). Hypomethylation of ras oncogenes in primary human cancers.. Biochem Biophys Res Commun.

[11] Ha PK, Califano JA (2006). Promoter methylation and inactivation of tumour-suppressor genes in oral squamous-cell carcinoma.. Lancet Oncol.

[12] Dickson MA, Hahn WC, Ino Y, Ronfard V, Wu JY (2000). Human keratinocytes that express hTERT and also bypass a p16(INK4a)-enforced mechanism that limits life span become immortal yet retain normal growth and differentiation characteristics.. Mol Cell Biol.

[13] Yamashita K, Upadhyay S, Osada M, Hoque MO, Xiao Y (2002). Pharmacologic unmasking of epigenetically silenced tumor suppressor genes in esophageal squamous cell carcinoma.. Cancer Cell.

[14] Tomlins SA, Rhodes DR, Perner S, Dhanasekaran SM, Mehra R (2005). Recurrent fusion of TMPRSS2 and ETS transcription factor genes in prostate cancer.. Science.

[15] Li LC, Dahiya R (2002). MethPrimer: designing primers for methylation PCRs.. Bioinformatics.

[16] Barsyte-Lovejoy D, Lau SK, Boutros PC, Khosravi F, Jurisica I (2006). The c-Myc oncogene directly induces the H19 noncoding RNA by allele-specific binding to potentiate tumorigenesis.. Cancer Res.

[17] Tsang WP, Kwok TT (2007). Riboregulator H19 induction of MDR1-associated drug resistance in human hepatocellular carcinoma cells.. Oncogene.

[18] Tsai JR, Chong IW, Chen YH, Yang MJ, Sheu CC (2007). Differential expression profile of MAGE family in non-small-cell lung cancer.. Lung Cancer.

[19] Laduron S, Deplus R, Zhou S, Kholmanskikh O, Godelaine D (2004). MAGE-A1 interacts with adaptor SKIP and the deacetylase HDAC1 to repress transcription.. Nucleic Acids Res.

[20] Foldi M, Stickeler E, Bau L, Kretz O, Watermann D (2007). Transketolase protein TKTL1 overexpression: A potential biomarker and therapeutic target in breast cancer.. Oncol Rep.

[21] Hu LH, Yang JH, Zhang DT, Zhang S, Wang L (2007). The TKTL1 gene influences total transketolase activity and cell proliferation in human colon cancer LoVo cells.. Anticancer Drugs.

[22] Krockenberger M, Honig A, Rieger L, Coy JF, Sutterlin M (2007). Transketolase-like 1 expression correlates with subtypes of ovarian cancer and the presence of distant metastases.. Int J Gynecol Cancer.

[23] Langbein S, Zerilli M, Zur Hausen A, Staiger W, Rensch-Boschert K (2006). Expression of transketolase TKTL1 predicts colon and urothelial cancer patient survival: Warburg effect reinterpreted.. Br J Cancer.

[24] Staiger WI, Coy JF, Grobholz R, Hofheinz RD, Lukan N (2006). Expression of the mutated transketolase TKTL1, a molecular marker in gastric cancer.. Oncol Rep.

[25] Zhang S, Yang JH, Guo CK, Cai PC (2007). Gene silencing of TKTL1 by RNAi inhibits cell proliferation in human hepatoma cells.. Cancer Lett.

[26] Volker HU, Scheich M, Schmausser B, Kammerer U, Eck M (2007). Overexpression of transketolase TKTL1 is associated with shorter survival in laryngeal squamous cell carcinomas.. Eur Arch Otorhinolaryngol.

[27] IGC (2005). expO (Expression Project for Oncology).. International Genetics Consortium.

[28] van der Bruggen P, Traversari C, Chomez P, Lurquin C, De Plaen E (1991). A gene encoding an antigen recognized by cytolytic T lymphocytes on a human melanoma.. Science.

[29] Duan Z, Duan Y, Lamendola DE, Yusuf RZ, Naeem R (2003). Overexpression of MAGE/GAGE genes in paclitaxel/doxorubicin-resistant human cancer cell lines.. Clin Cancer Res.

[30] Monte M, Simonatto M, Peche LY, Bublik DR, Gobessi S (2006). MAGE-A tumor antigens target p53 transactivation function through histone deacetylase recruitment and confer resistance to chemotherapeutic agents.. Proc Natl Acad Sci U S A.

[31] Nagao T, Higashitsuji H, Nonoguchi K, Sakurai T, Dawson S (2003). MAGE-A4 interacts with the liver oncoprotein gankyrin and suppresses its tumorigenic activity.. J Biol Chem.

[32] Yang B, O'Herrin S, Wu J, Reagan-Shaw S, Ma Y (2007). Select cancer testes antigens of the MAGE-A, -B, and -C families are expressed in mast cell lines and promote cell viability in vitro and in vivo.. J Invest Dermatol.

[33] Yang B, O'Herrin SM, Wu J, Reagan-Shaw S, Ma Y (2007). MAGE-A, mMage-b, and MAGE-C proteins form complexes with KAP1 and suppress p53-dependent apoptosis in MAGE-positive cell lines.. Cancer Res.

[34] Suzuki T, Yoshida K, Wada Y, Hamai Y, Sentani K (2007). Melanoma-associated antigen-A1 expression predicts resistance to docetaxel and paclitaxel in advanced and recurrent gastric cancer.. Oncol Rep.

[35] De Castro Valente Esteves LI, De Karla Cervigne N, Do Carmo Javaroni A, Magrin J, Kowalski LP (2006). H19-DMR allele-specific methylation analysis reveals epigenetic heterogeneity of CTCF binding site 6 but not of site 5 in head-and-neck carcinomas: a pilot case-control analysis.. Int J Mol Med.

[36] Jelinic P, Stehle JC, Shaw P (2006). The testis-specific factor CTCFL cooperates with the protein methyltransferase PRMT7 in H19 imprinting control region methylation.. PLoS Biol.

[37] Giannoukakis N, Deal C, Paquette J, Goodyer CG, Polychronakos C (1993). Parental genomic imprinting of the human IGF2 gene.. Nat Genet.

[38] Bliek J, Terhal P, van den Bogaard MJ, Maas S, Hamel B (2006). Hypomethylation of the H19 gene causes not only Silver-Russell syndrome (SRS) but also isolated asymmetry or an SRS-like phenotype.. Am J Hum Genet.

[39] Prawitt D, Enklaar T, Gartner-Rupprecht B, Spangenberg C, Lausch E (2005). Microdeletion and IGF2 loss of imprinting in a cascade causing Beckwith-Wiedemann syndrome with Wilms' tumor.. Nat Genet.

[40] Prawitt D, Enklaar T, Gartner-Rupprecht B, Spangenberg C, Oswald M (2005). Microdeletion of target sites for insulator protein CTCF in a chromosome 11p15 imprinting center in Beckwith-Wiedemann syndrome and Wilms' tumor.. Proc Natl Acad Sci U S A.

[41] Sparago A, Cerrato F, Vernucci M, Ferrero GB, Silengo MC (2004). Microdeletions in the human H19 DMR result in loss of IGF2 imprinting and Beckwith-Wiedemann syndrome.. Nat Genet.

[42] Issa JP (2004). CpG island methylator phenotype in cancer.. Nat Rev Cancer.

[43] Toyota M, Ahuja N, Ohe-Toyota M, Herman JG, Baylin SB (1999). CpG island methylator phenotype in colorectal cancer.. Proc Natl Acad Sci U S A.

[44] Kholmanskikh O, Loriot A, Brasseur F, De Plaen E, De Smet C (2008). Expression of BORIS in melanoma: lack of association with MAGE-A1 activation.. Int J Cancer.

[45] Nguyen P, Bar-Sela G, Sun L, Bisht KS, Cui H (2008). BAT3 and SET1A form a complex with CTCFL/BORIS to modulate H3K4 histone dimethylation and gene expression.. Mol Cell Biol.

[46] Bolli M, Schultz-Thater E, Zajac P, Guller U, Feder C (2005). NY-ESO-1/LAGE-1 coexpression with MAGE-A cancer/testis antigens: a tissue microarray study.. Int J Cancer.

[47] Cho HJ, Caballero OL, Gnjatic S, Andrade VC, Colleoni GW (2006). Physical interaction of two cancer-testis antigens, MAGE-C1 (CT7) and NY-ESO-1 (CT6).. Cancer Immun.

[48] Eden A, Gaudet F, Waghmare A, Jaenisch R (2003). Chromosomal instability and tumors promoted by DNA hypomethylation.. Science.

[49] Herman JG, Graff JR, Myohanen S, Nelkin BD, Baylin SB (1996). Methylation-specific PCR: a novel PCR assay for methylation status of CpG islands.. Proc Natl Acad Sci U S A.

[50] Vatolin S, Abdullaev Z, Pack SD, Flanagan PT, Custer M (2005). Conditional expression of the CTCF-paralogous transcriptional factor BORIS in normal cells results in demethylation and derepression of MAGE-A1 and reactivation of other cancer-testis genes.. Cancer Res.

[51] Benjamini Y, Hochberg Y (1995). Controlling the false discovery rate: a practical and powerful approach to multiple testing.. J R Stat Soc B.

