# Supplementary figures and images for: Coordinated Activation of Candidate Proto-Oncogenes and Cancer Testes Antigens via Promoter Demethylation in Head and Neck Cancer and Lung Cancer
Source: PLoS One. 2009 Mar 23;4(3):e4961. doi: 10.1371/journal.pone.0004961 (PMC2654921; doi:10.1371/journal.pone.0004961)

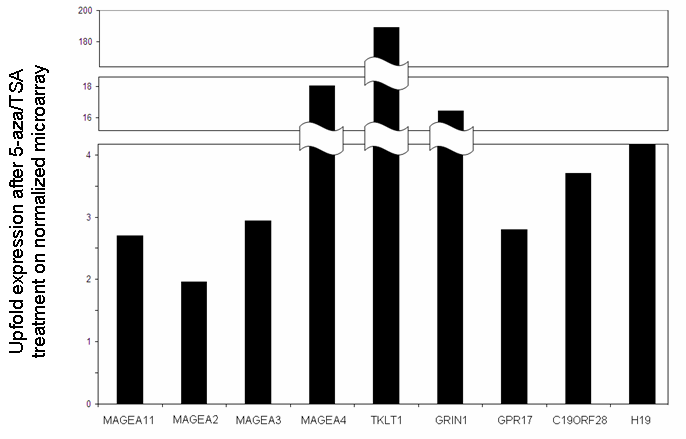

Supplement: Figure S1 — Upfold regulation of mRNA expression in treated minimally-transformed cell lines measured by Affymetrix U133 Plus 2.0. (0.05 MB TIF) [file pone.0004961.s001.tif]

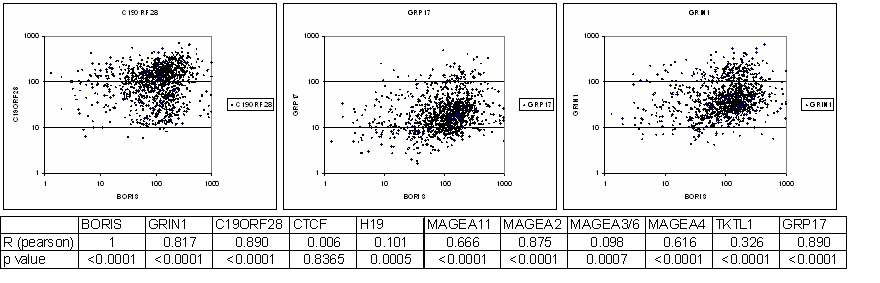

Supplement: Figure S2 — BORIS correlates with gene expression in all cancers (using the expO cohort of 1041 human cancers of various tumor sites and histologies). Shown are microarray median-normalized expression of our targets compared to BORIS expression in 1041 human cancers. (0.03 MB TIF) [file pone.0004961.s002.tif]
